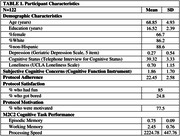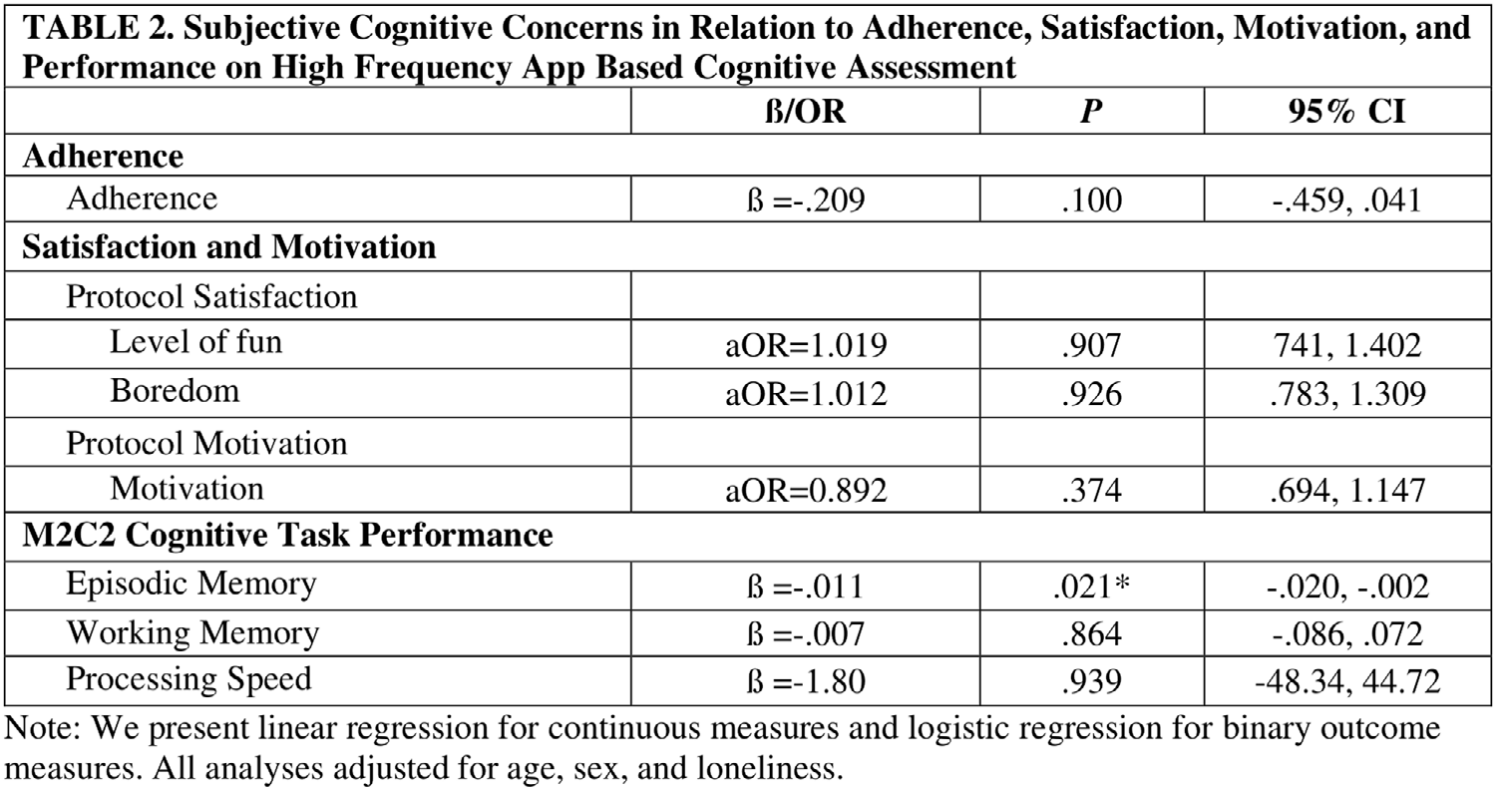# Subjective Cognitive Concerns and High Frequency Mobile App Based Cognitive Assessment in Older Adults: Relationship to Adherence, Satisfaction, and Cognitive Performance

**DOI:** 10.1002/alz.091669

**Published:** 2025-01-03

**Authors:** Caroline O Nester, Alyssa N. De Vito, Zachary J. Kunicki, Jennifer Strenger, Karra D Harrington, Nelson A. Roque, Martin J. Sliwinski, Louisa I. Thompson

**Affiliations:** ^1^ Alpert Medical School of Brown University, Providence, RI USA; ^2^ Butler Hospital Memory and Aging Program, Providence, RI USA; ^3^ The Pennsylvania State University, University Park, PA USA; ^4^ Pennsylvania State University, State College, PA USA

## Abstract

**Background:**

Subjective cognitive concerns (SCC) are possibly one of the earliest clinical symptoms of dementia. There is growing interest in applying mobile app‐based assessment to remotely screen for cognitive status in preclinical dementia, but the relationship between SCC and relevant mobile assessment metrics remains unclear. To address this gap, we characterize the relationship between SCC and adherence, satisfaction, and performance on digital cognitive assessment in cognitively unimpaired older adults.

**Method:**

Participants (N = 122, Table 1) completed 8 assessment days using Mobile Monitoring of Cognitive Change (M2C2), an app‐based testing platform, with brief daily sessions within morning, afternoon, and evening time windows (24 total testing sessions). M2C2 includes working memory, processing speed, and episodic memory tasks. Participants provided feedback about their satisfaction and motivation related to M2C2. SCC was assessed via the Cognitive Function Instrument (CFI). Regression analyses evaluated the association between SCC and adherence, satisfaction, and performance on M2C2, controlling for age, sex, and loneliness (UCLA Loneliness Scale). Linear‐mixed effects models evaluated whether SCC predicted M2C2 subtest performance longitudinally over the 8‐day testing period, controlling for covariates.

**Result:**

SCC was not associated with adherence to M2C2, app satisfaction, or protocol motivation. SCC endorsement significantly predicted worse overall episodic memory performance (ß = ‐0.011, p = .021, 95% CI ‐0.02, ‐0.00), but was not associated with overall working memory or processing speed (Table 2). A trend was revealed between SCC and slow processing speed at day 1. There was a significant interaction between SCC and processing speed over the 8‐day period, such that SCC was associated with initially slow, then progressively faster processing speed.

**Conclusion:**

SCCs are associated with objective cognitive performance on mobile app‐based assessments, but do not impact metrics related to protocol engagement (e.g., adherence, satisfaction, and motivation), suggesting that mobile app assessments may be sensitive to early neurodegenerative changes. Results provide reassurance that SCCs do not impact the feasibility of mobile app‐based assessments in older adult samples. Future research in larger, more diverse samples which follow cognitive functioning longitudinally will be essential next steps to understand the utility of SCC to predict cognitive decline via mobile app.